# Prevalence of BRCA mutations among hereditary breast and/or ovarian cancer patients in Arab countries: systematic review and meta-analysis

**DOI:** 10.1186/s12885-019-5463-1

**Published:** 2019-03-21

**Authors:** Khadiga Abdulrashid, Nour AlHussaini, Wifag Ahmed, Lukman Thalib

**Affiliations:** Department of Public Health, College of Health Sciences, Qatar University, PO Box 2713, Doha, Qatar

**Keywords:** BRCA mutations, Familial breast cancer, Familial ovarian cancer, Arab countries, Systematic review, meta-analysis

## Abstract

**Background:**

To systematically assess the prevalence of BRCA1 and BRCA2 gene mutations in women with Hereditary Breast and/or Ovarian Cancer (HBOC) in Arab countries and to describe the variability in the BRCA gene mutations in different regions of the Arab world.

**Methods:**

Observational studies reporting prevalence of BRCA mutations from 22 Arab countries were systematically searched in databases including PUBMED, EMBASE, Web of Science, and Google Scholar. Two reviewers independently screened the studies and extracted data and assessed the risk of bias. Hoy’s risk of Bias tool was used to assess the biases in individual studies. Due to substantial heterogeneity, pooled weighted estimates were calculated using Quality Effect Models (QEM) that adjust for bias, while the Random Effect Models (REM) estimates served as the sensitivity estimates.

**Results:**

Fourteen studies reporting prevalence of BRCA were included. The pooled estimate of BRCA among HBOC was 20% (95% CI: 7–36%). Subgroup analysis including only those with low risk of bias provided an estimate of 11% (95% CI: 1–27%). Levant region had higher prevalence 28% (95% CI: 11–49%) compared to Arabian Gulf region and North Africa but differences are not statistically significant, when tested using Z-test for proportions.

**Conclusion:**

Given the pooled estimates vary widely with substantial heterogeneity, larger, well-designed studies are warranted to better understand the frequency and the impact of BRCA gene mutations among Arab women.

**Trial registration:**

International Prospective Register of Systematic Reviews (PROSPERO) registration number: CRD42018095905.

## Background

Hereditary Breast and/or Ovarian cancer (HBOC) is an autosomal dominant cancer, that produce higher than normal levels of breast cancer and ovarian cancer in genetically related families [1]. About 5–10% of all breast cancer cases [2] and more than 23% of all ovarian cancers [3] are thought to be hereditary. BRCA genes have been identified to be the most commonly linked germ line mutations [4]. Harmful mutations in the *BRCA1* and *BRCA2* genes can produce very high rates of breast and ovarian cancer and increases the risk of developing breast cancer by up to 85% and the ovarian cancer by up to 54% [3].

However, BRCA frequencies are known to vary between populations. For instance, the prevalence of BRCA1 in Japan were reported to be as low as 2.6% while in the USA it can be as high as 11.1% [30]. Understanding population specific BRCA gene distributions can be helpful in developing appropriate risk assessment strategies that can help reduce the risk of developing cancers. It is also important in developing cost-effective strategies for genetic testing for BRCA mutations [5]. In terms of prognosis breast or/and ovarian cancer patients with family history of the disease with one or more of family members differs from sporadic cancers that occur without any hereditary link [6].

However, there is paucity of high quality data on the epidemiology of BRCA mutations in Arab populations, despite the fact that the breast cancer is the leading cause of all cancer deaths among Arab women [36]. The breast cancer also accounts for about 14 to 42% of all female cancers in the Arab world [37]. For instance, in Lebanon the breast cancer is leading in cancer incidences, with about 37.6% of all new female cancer cases diagnosed during the period of 2004–2010 [38]. A study in Yemen showed that breast cancer accounted 22% from all cancers cases [39]. Similar incidences were reported from Oman, where the breast cancer accounts for 25% of all cancer cases [40]. Given that breast cancer is the most commonly diagnosed cancer among Arab women [7], understanding the genetic role can help deal with the diseases better. Moreover, breast and ovarian cancer patients (BOC) are also diagnosed as early onset cases in the Arab countries. This led some to suggest that there exist variant mutations of BRCA genes among Arabs [8].

To the best of our knowledge, there is no aggregated data on BRCA gene distribution among Arab women. To this effect, we aimed to quantify the prevalence of BRCA1 and BRAC2 gene mutations and to explore the geographic variability of these gene mutations within the Arab world. Given the clinical implications in terms of diagnosis, risk stratifications, and management of breast or/and ovarian cancer, quantifying the extent to which these gene mutations prevail in the Arab populations can be highly valuable.

## Methods

### Protocol and registration

This present study was conducted following the recommendations provided by the PRISMA [9] and MOOSE guidelines [10]. This study was registered with the International Prospective Register of Systematic Reviews (PROSPERO) and the registration number is: CRD42018095905.

### Inclusion and exclusion criteria

The following inclusion criteria were used to identify eligible studies: peer reviewed studies published in English or Arabic; cohort studies, cross-sectional, case-series or registry based studies, providing sufficient data to compute the prevalence of either BRCA1 or BRCA2 gene mutations in the breast and / or ovarian cancer patients in the Arab world.

Studies were excluded if they were confined mainly to the carriers of BRCA mutations without including other cancer patients. Those studies that has no information on family history of disease were also excluded as there is no way of quantifying HBOC. Likewise, experimental studies that were evaluating the effect of an intervention on HBOC patients were also excluded as computing prevalence in these studies were not possible. Additionally, studies reporting on BRCA1/2 genes mutations in cancers other than breast or ovarian cancers were also excluded.

### Information sources and databases

The electronic databases Web of Science, PubMed/Medline and EMBASE were searched to identify the primary studies up to April 11, 2018. Further search included Google Scholar search to identify any relevant studies as well hand search of the reference lists of the relevant articles. Although, there were no systematic reviews or meta-analyses from this region, we searched for all review articles reporting on the BRCA mutations form other parts of the world to identify any relevant studies from Arab countries. Only those published in English and Arabic were included, and the duplicates studies were removed using EndNote software.

### S**earch strategy**

Studies from all 22 Arab member states of the Arab league [11] were included. We used the following terms to be in the “Titles” or “Abstracts: [BRCA],” and [“Arabs,” or “Qatar,” or “Kuwait,” “Oman,” or “Iraq,” or “Saudi Arabia,” or “Bahrain,” or “United Arab Emirates,” or “Yamen,” “Syria,” or “Lebanon,” or “Jordan,” or “Palestine,” or “Jerusalem,” or “Gaza,” or “Egypt,” or “Libya,” or “Sudan,” or “Tunisia,” or “Morocco,” or “Algeria,” or “Somalia,” or “Comoros,” or “Djibouti,” or “Mauritania.”] and any other variant names for any of these countries.

### Study selection

Two review authors independently assessed titles and abstracts of all citations retrieved by the search for relevance against the inclusion criteria. Then the full-text versions of studies considered potentially eligible were retrieved. The same two authors independently assessed the full papers for eligibility, with disagreements resolved through input by a third author [12]. When the eligibility of a study was unclear, we attempted to contact study authors.

### Data extraction

Two authors independently extracted data from eligible studies and cross-checked for accuracy and agreement by the third author. Data extraction using a standardized form included variables such as author, year of publication, country, city, study design, type of disease (BC/OC), age at diagnosis, mutation type, mutation test used, overall sample size and sample size of HBOC subset patients (Table 1).

**Table 1:** Characteristics of Studies Included

Author	Year	Country	City	Design	HBOC/HBC	Age at Diagnosis (range or mean)	Mutation type	Test type	Sample size	Sample size of HBOC
Abdel-Razeq	2018	Jordan	–	Pilot study	HBC^b^	22–75	Deleterious Suspected-deleterious	BART^1^	100	84
Bu	2016	Saudi Arabia	–	Cross-sectional	HBC	41.9	Deleterious	PCR^2^, CS^3^/SS^4^, and TCS^5^	818	60
Bujassoum	2017	Qatar	–	Retro^a^	HBC	23–68	Deleterious	MLPA^6^	82	82
Cherbal	2015	Algeria	High Plains	Cross-sectional	HBOC^c^	< 50	Deleterious VUS	PCR^2^	192	192
El Saghir	2015	Lebanon	Beirut	Cross-sectional	HBC	40.8	Deleterious VUS	MLPA^6^	250	74
Ibrahim	2010	Egypt	Alexandria	Registry data	HBC	43.5	Deleterious	SSCP^7^ HAC^8^	60	39
Jalkh	2012	Lebanon	–	Cross-sectional	HBC	41	Deleterious VUS Polymorphisms	Fluorescent DS^9^	72	72
Kadouri	2007	Palestine	East Jerusalem	Cross-sectional	HBC	–	Deleterious VUS Polymorphisms	DHPLC^10^	31	10
Laarabi	2017	Morocco	North-East morocco (Rabat, Fes, Oujda)	Cross-sectional	HBOC	–	Deleterious	CSS^11^ NGS^12^ TS^13^	122	122
Laraqui	2013	Morocco	Rabat	Retro	HBC	–	Deleterious	DS^14^	121	19
Mahfoudh	2012	Tunisia	Sousse	Cross-sectional	HBC	29–65	Deleterious VUS Polymorphisms	DS^15^	24	24
Riahi	2016	Tunisia	–	Cross-sectional	HBC	–	Deleterious	Logistic regression model (studied three studies)	92	92
Tazzite	2012	Morocco	Casablanca	Case series	HBOC	25–60	Deleterious	DS^15^	40	34
Uhrhammer	2008	Algeria	Algiers	Pilot study	HBC	15–52	Deleterious VUS Polymorphisms	PCR^2^	64	13

^a^Retro: retrospective cohort study

^b^HBC: hereditary breast cancer

^c^HBOC: hereditary breast and/or ovarian cancer

^1^BART: Comprehensive BRACAnalysis and BRACAnalysis rearrangement test

^2^PCR: Polymerase chain reaction

^3^CS: Capture sequencing

^4^SS: Sanger sequencing

^5^TCS: Targeted capture sequencing

^6^MLPA: Multiple ligation dependent probe amplification

^7^SSCP: single strand conformation polymorphism assay

^8^HAC: Heteroduplex assay confirmation

^9^Fluorescent direct sequencing of the entire coding region and intronic sequence flanking each exon

^10^DHPLC: denaturing high performance liquid chromatography

^11^CSS: Conventional individual exon-by-exon Sanger sequencing

^12^NGS: Next generation sequencing

^13^TS: Target screening for exon 10 in BRCA2

^14^Direct sequencing of all coding exons and flanking intron sequences of the BRCA1 gene

^15^DS: Direct Sequencing

^16^Direct sequencing

### Methodological quality assessment

We used the risk of bias assessment tool developed by Hoy et al. [13] to evaluate the quality of individual studies. The tool has 10 items: four items were related to external validity in terms of how well study sample represent the study population and remaining six items were related to internal validity of the studies. Total quality scores for each study was obtained by summing the binary response scores (Yes = 1 or No =0) that ranged from 0 to 10. As per Hoy et al. [13], scores above 7 indicated low risk of bias while a score between 6 to 7 indicated moderate risk of bias. Those studies scoring less than 6 were considered to have high risk of bias.

### Syntheses of results

We pooled the quantitative data using Meta-XL version 5.3 [14]. We reported the pooled prevalence and 95% confidence intervals (CI) and explored the robustness of meta-analyses using appropriate meta-analytical models based on the level of heterogeneity. Statistical heterogeneity were assessed using Cochrane Q statistic and I-squared (I^2^) statistics [15].

In the absence of heterogeneity, the Fixed Effect Models (FEM) were used. When substantial heterogeneity is encountered, the Quality Effects Models (QEM) were used to pool the prevalence [16]. Significant heterogeneity is indicated by a significant *P*-value of the Cochrane-Q test or an I^2^ statistic value that is higher 50%. We used the quality scores computed by ROB assessment tool to fit the bias adjusted QEM. Additionally, Random Effect Models (REM) were used to obtain sensitivity estimates when substantial heterogeneity is encountered. We further investigated the sources of heterogeneity using subgroup analysis. We used a-priori defined test to compare the pooled prevalence in three different regions of the Arab world: Levant, North Africa and Arabian Gulf Co-operation Countries (GCC) using Z- tests for proportions.

## Results

### Selection of studies

**Fig. 1:**
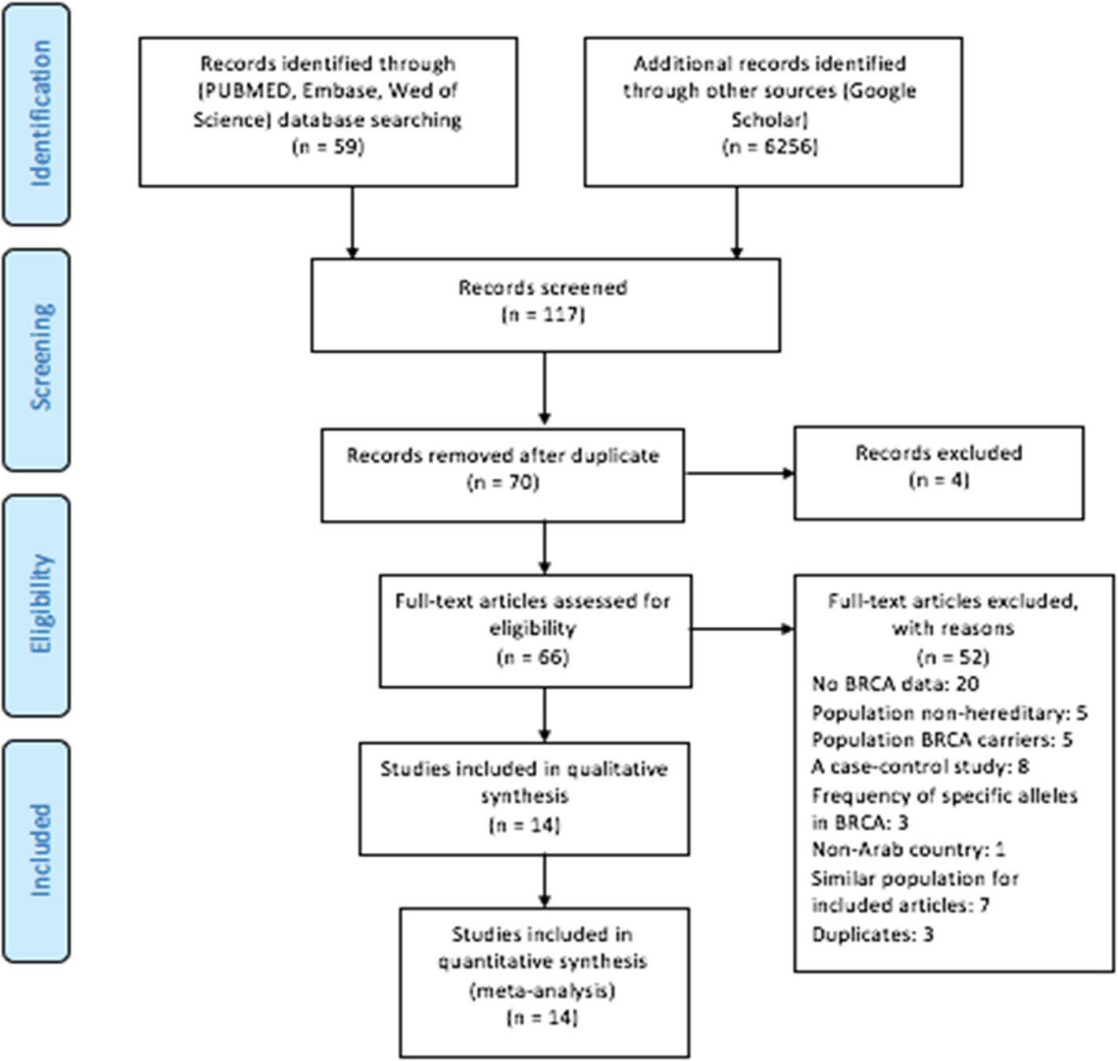
Flowchart representing the process of screening and selection of eligible studies, based on Preferred Reporting Items for Systematic Reviews and Meta-Analyses (PRISMA) guidelines

The database search generated a total of 6315 records (Fig. 1). We excluded 6249 studies and reasons for exclusions are listed in the Fig. 1. We assessed the full text of 66 studies for their eligibility. Three authors independently read through the full texts and identified those eligible for the review based on a-priori inclusion criteria. Only 14 publications were found to be eligible and included in this study.

### Study characteristics

The 14 studies selected were all peer reviewed full length publications form nine different Arab countries. All these 14 articles were published in English and published during the period of 2007 and 2018. They reported on the prevalence of BRCA1 and / or BRCA2 mutations among breast or/ and ovarian cancer patients. Of these, eight were conducted in North African countries, four from Levant, and two from GCC. There were three studies from Morocco: one each from North-east Morocco [27], Casablanca [22] and Rabat [4]. Two studies each from the following countries. Algeria [23, 25], Lebanon [7, 26], and Tunisia [20, 21]. Single studies from each of the following countries: Qatar [17], Saudi Arabia [5], Jordan [24], Palestine [19], and Egypt [18].

Sample sizes of the included studies ranged between10 to 192, and total number of patients pooled was 917. Of these, 11 studies included Hereditary Breast Cancer (HBC) patients, three had Hereditary Breast and/or Ovarian Cancer (HBOC) patients, and a single study included only Heredity Ovarian Cancer (HOC) patients. Seven studies investigated both BRCA1 and BRCA2 gene mutations, six studies had data on only BRCA1 gene mutation and one study was exclusively on BRCA2 mutations. There were variation in the methods used to detect BRCA mutations. Three studies used direct sequencing, while others used varying types of methods.

### Risk of Bias

**Fig. 2:**
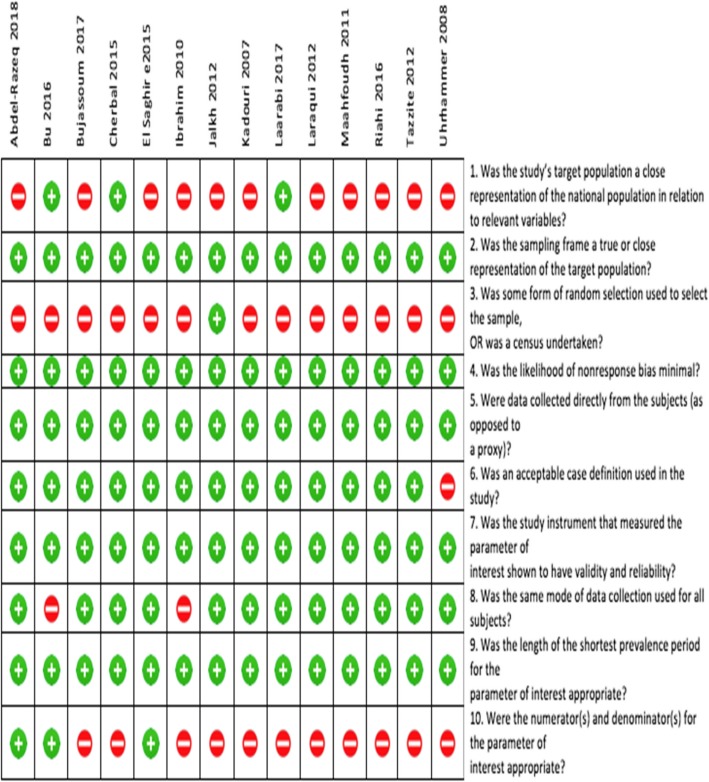
Risk of bias plot that shows the methodological quality assessment of the 14 studies included

Of the 14 articles included in our study, eight studies were found to have moderate risk of bias [4, 17–23], and six had lower risk of bias [5, 7, 24–27]. In term of the external validity components, only three articles were considered to have a good external validity [5, 25, 27] (See Fig. 2).

### Quantitative synthesis

**Fig. 3:**
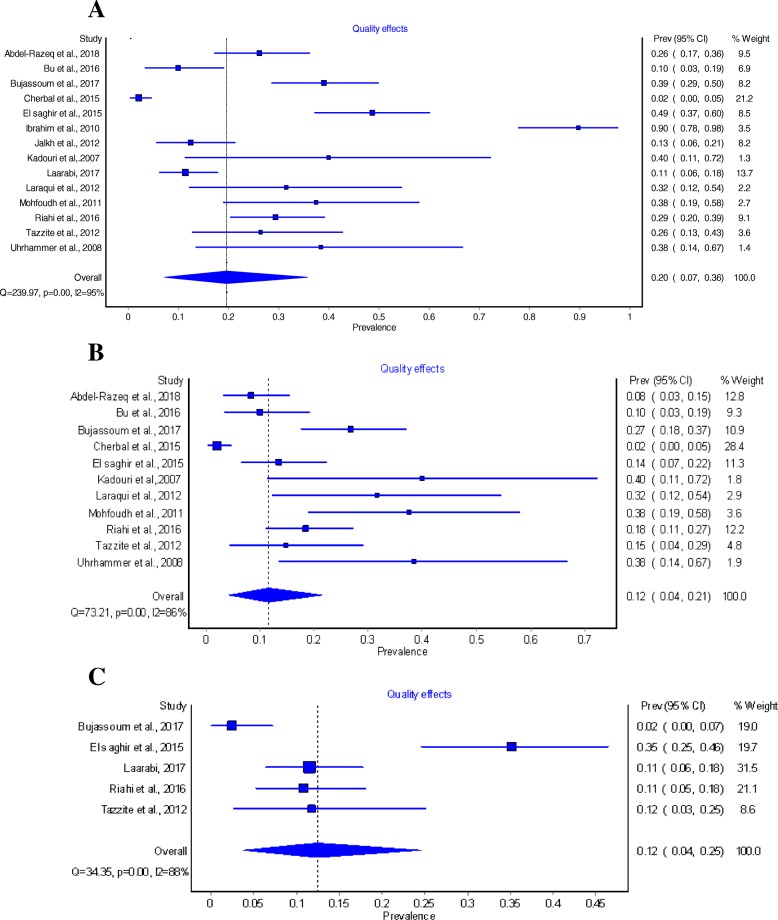
**a** Pooled prevalence of any BRCA mutations among hereditary population obtained using Quality Effect Model (QEM). **b** Pooled prevalence of BRCA1 mutations among hereditary sample obtained using Quality Effect Model (QEM). **c** Pooled prevalence of BRCA2 mutations among hereditary sample obtained using Quality Effect Model (QEM)

From studies reported total BRCA mutation cases, the overall pooled prevalence of BRCA among breast and/or ovarian cancer based on QEM was 20% (95% CI: 7–36%) [4, 5, 7, 17–27] (Fig. 3a). The pooled prevalence of BRCA1 mutation among hereditary breast and/ or ovarian cancer was 12% (95% CI: 4–21%) (Fig. 3b) [4, 5, 7, 17, 19–25]. The estimated pooled prevalence of BRCA2 was 12% (95% CI: 4–25%) based on five studies reported such mutation in the HBOC population [7, 17, 21, 22, 27] (Fig. 3c). Further, six studies reported prevalence of BRCA mutation among non- hereditary sample [4, 5, 7, 18, 22, 23]. The pooled prevalence in this population was estimated to be 10% (95% CI: 0–28%).

Of all 14 studies included, 13 reported prevalence of BRCA among breast cancer patients and the pooled prevalence was 21% (95% CI: 6–40%) [4, 5, 7, 17, 19–26]. Of this, the prevalence of BRCA1 mutation alone among breast cancer patients was 11% (95% CI: 4–21%) [4, 5, 7, 17, 19–25]. Likewise, the prevalence of BRCA2 mutations among breast cancer patients was 17% (95% CI: 9–27%) [7, 17, 21, 22, 24]. Only one study reported the prevalence of BRCA1 mutations among ovarian cancer patients which was in Algeria [25] with a prevalence of 1% (95% CI: 0–2%).Three studies [17, 22, 27] out of 14 studies report a prevalence of BRCA1 or BRCA2 among both Breast and Ovarian Cancer patients, the pooled prevalence was 9% (95% CI: 0–25%).

All the estimates provided above are QEM based to adjust for bias in individual studies. However, the Random Effect Model (REM) is a commonly used and is recommended by Cochrane group as the choice of model when there is substantial heterogeneity between studies included in a meta-analysis [12]. We therefore used REM as a sensitivity method to compare the QEM estimates. Table 2 shows a comparison between QEM and REM estimates, which were not much different.

**Table 2:** Comparison between Quality Effect Model and the Random Effect Model results

		Quality effect model			Random effect model		
		No. of studies (patients)	Prevalence (CI 95%)	I^2^%	No. of studies (patients)	Prevalence (CI 95%)	I^2^%
All eligible studies		14 (917)	20% (7–36%)	94.6%	14 (917)	63% (17–44%)	94.6%
Risk of Bias							
	Low ROB	6 (604)	11% (1–27%)	94.7%	6 (604)	66% (4–31%)	94.7%
	Moderate ROB	8 (313)	40% (21–60%)	87.1%	2 (313)	63% (26–59%)	87.1%
	Studies with good external validity	3 (374)	5% (0–14%)	85.5%	3 (374)	49% (1–16%)	85.5%
Gene mutations							
	Both BRCA1 and BRCA2	7 (477)	34% (18–52%)	93.2%	7 (477)	78% (22–56%)	93.2%
	BRCA1	11 (684)	19% (7–36%)	92.8%	11 (684)	59% (15–42%)	92.8%
	BRCA2	6 (488)	27% (16–40%)	87.4%	6 (488)	75% (18–42%)	87.4%
Target population							
	HBOC	3 (348)	7% (0–20%)	91.8%	3 (348)	55% (0–25%)	91.8%
	HBC	11 (569)	32% (18–47%)	90.8%	10 (550)	65% (23–50%)	90.8%
	HOC	1 (192)	2% (0–5%)	0	1 (192)	33% (0–5%)	0
Geographic Location							
	Levant	4 (240)	28% (11–49%)	87.9%	4 (240)	63% (13–49%)	87.9%
	GCC	2 (142)	22% (0–58%)	93.9%	2 (142)	24% (0–56%)	93.9%
	North Africa	8 (535)	16% (0–43%)	96.2%	8 (535)	64% (11–54%)	96.2%
Sample size							
	< 100	12 (603)	29% (16–44%)	89.9%	12 (603)	62% (21–48%)	89.9%
	≥100	2 (314)	5% (0–16%)	91.4%	2 (314)	52% (0–17%)	91.4%
Year of recruitment							
	≤2011	10 (467)	25% (8–47%)	93.1%	10 (467)	60% (17–52%)	93.1%
	2012–2018	5 (572)	13% (1–31%)	95.6%	5 (572)	71% (5–37%)	95.6%
Type of mutation							
	Deleterious	8 (532)	26% (11–44%)	93.6%	8 (532)	71% (17–49%)	93.6%
	Deleterious and VUS	2 (266)	10% (0–100%)	98.8%	2 (266)	73% (0–85%)	98.8%
	Deleterious, VUS, and Polymorphisims	4 (119)	20% (4–43%)	72.4%	4 (119)	46% (7–57%)	85.0%

We repeated the primary analysis of overall pooled prevalence of BRCA among hereditary breast or/and ovarian cancer by only including the low risk of bias studies to estimate the prevalence of high quality studies [5, 7, 24–27]. The pooled prevalence of BRCA mutations among the high quality studies were estimated to be 11% (95CI: 1–27%). This compared to the overall pooled prevalence including all studies was lower and much more comparable with the global estimates.

Quality effect model (QEM) used for subgroup analysis from different geographical locations in GCC, Levant and North Africa to understand any geographic variations in the prevalence of BRCA mutations among hereditary breast or/and ovarian cancer patients. The highest pooled prevalence of BRCA mutations was estimated in Levant region the pooled prevalence was 28% (95% CI: 11–49%) followed by the Gulf region where the pooled prevalence was 22% (95% CI: 0–58%). Comparable prevalence in the North African region was lower than other regions, as it was estimated to be 16% (95%CI: 0–44%). These estimates did not differ statistically based on Z- test for proportions.

### Heterogeneity assessment

Significant heterogeneity was encountered throughout the analysis as indicated by the *p* values of the Cochrane Q statistics and I^2^ statistics values. For instance, the studies [4, 5, 7, 17–27] included to pool the prevalence for BRCA mutations had a Cochran Chi-square p value of < 0.001 and I^2^ of 95%. Likewise, there were significant heterogeneity across the studies used to obtain the pooled prevalence for BRCA1 among total HBOC (Chi-square = 69.2, *p*-value< 0.001, I^2^ = 86%) [4, 5, 7, 17, 19–25]. For BRCA2 heterogeneity between the studies were also substantial (Chi-square = 23.5, p-value < 0.001, I^2^ = 79.7%) [7, 17, 21, 22, 27].

For total BRCA mutations among general population, there was a considerable heterogeneity across studies (Chi-square = 70, *P* value = 0.001, I- square = 94.3%) and the pooled prevalence was 35% (95% %CI: 16–55%). The pooled prevalence of BRCA1 mutation among Breast Cancer Patients was 11% (95% CI: 4–21%) [4, 5, 7, 17, 19–25], and there was a substantial heterogeneity across the studies (Chi-square = 79.3, P value < 0.001, I-square = 87.38%). As for the prevalence of BRCA 2 mutation among Breast Cancer patients, data was reported from 5 studies [7, 17, 21, 22, 24]. Where the pooled prevalence was 17% (95% CI: 9–27%), and significant heterogeneity was found (Chi-square = 18.5, P value = 0.001, I square = 78.39%). Only one study [25] reported BRCA1 among ovarian cancer and no studies reported BRCA2 among ovarian cancer patients, so heterogeneity was not assessed. For BRCA1 or/and BRCA2 among patients with both Breast and Ovarian cancer, the pooled prevalence was 9% (95% CI: 0–25%) [17, 22, 27]. For heterogeneity, there was large heterogeneity detected (Chi-square = 19, P value < 0.001, I-square = 89.4%).

### Publication Bias

**Fig. 4:**
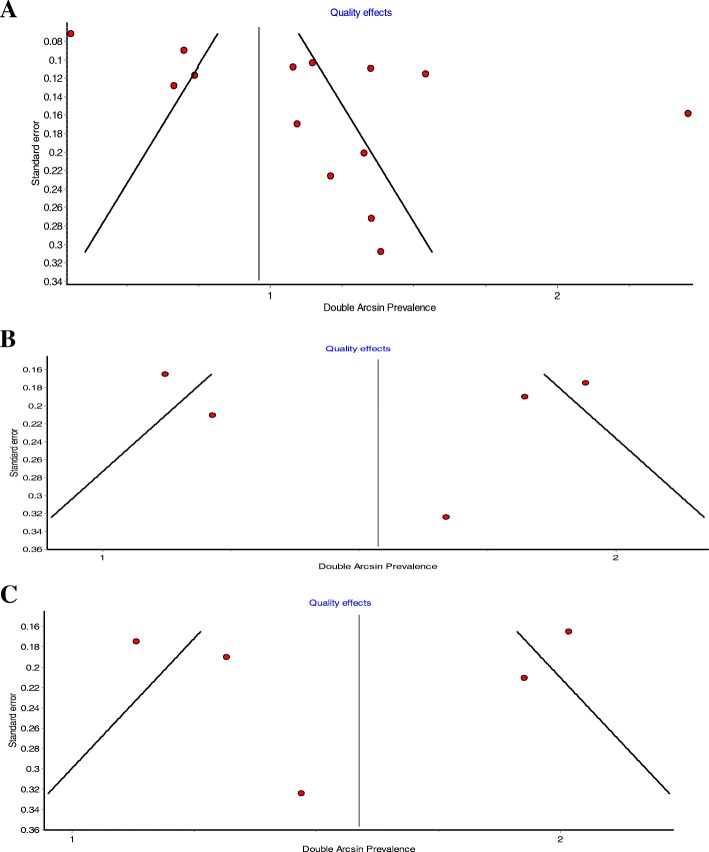
**a** Funnel plot assessing the publication bias in pooling the prevalence of any BRCA mutation. **b** Funnel plot assessing the publication bias in pooling the prevalence of BRCA1 mutation. **c.** Funnel plot assessing the publication bias in pooling the prevalence of BRCA2 mutation

Publication bias was investigated using classical funnel plot, where an asymmetry indicated potential publication bias (Fig. 4a, b and c). As for BRCA1 and BRCA2 mutations estimates in hereditary patients, funnel plots showed a potential publication bias. However, Hunter et al. [28] suggested that conventional funnel plots, that assess publication bias, are inaccurate for prevalence meta-analysis. We are unsure about the asymmetry observed in our study if it is true reflection of publication bias or simply an over-estimate as argued by Hunter et al. [28].

## Discussion

This study included 14 eligible studies from nine Arab countries, to systematically review the prevalence of BRCA mutations. The study revealed that one in five hereditary breast or/and ovarian cancer patients in the Arab region are likely to have BRCA mutations. The prevalence BRCA2 were more common (17%) than BRCA1 mutations (11%) in the region. The prevalence of BRCA mutations were also noted to vary considerably from other populations [31–35]. For instance, prevalence of BRCA gene mutations in India, Japan, Hispanics in USA, and Spain were substantially higher than that of the Arab populations, whilst Iran, Mexico, Sweden, Germany, Australia and Turkey reported significantly lower prevalence (Table 3).

**Table 3:** Comparison between the pooled prevalence in Arab countries with other regions around the world

Author (year)	Country	Sample size	BRCA mutation prevalence	z-score	*P*-value
Keshavarzi et al. (2011)	Iran	85	7%	15.429	< 0.0001
Vaca-Panigua et al. (2012)	Mexico	39	7.7%	13.97	< 0.0001
Loman et al. (2001)	Sweden	234	8.9%	11.81	< 0.0001
Hamann et al. (2003)	Germany	23	13%	6.30	< 0.0001
Alsop et al. (2012)	Australia	1001	14.1%	5.134	< 0.0001
Yazici et al. (2000)	Turkey	53	15.1%	4.144	< 0.0001
Sharma et al., (2014)	Kansas, USA	207	15.4%	3.86	0.0001
Han et al. (2013)	Korea	775	21.7%	1.25	0.2117
Hedau et al. (2004)	India	24	25%	3.497	= 0.0005
Sugano et al. (2008)	Japan	135	26.7%	4.59	< 0.0001
Weitzel et al. (2005)	Hispanics in USA	110	30.9%	7.14	< 0.0001
Hoya et al. (2001)	Spain	102	43%	14.07	< 0.0001

There were variability in the prevalence of BRCA mutations between different areas of the Arab region. Such differences might be explained by biological differences in the BRCA gene prevalence [23], varying age at onset in the study population with varying proportion of younger population in different parts of the Arab world. This was further amplified by the fact that the diagnosis at a young age is an indication for the referral for BRCA testing. Studies have also suggested that there is a variation of BRCA prevalence among different ethnicities [41], while others found that the prevalence of BRCA mutations is the same across different ethnicities [42]. Family history of breast or ovarian cancer could be a significantly strong predictor for being a carrier of BRCA gene mutations [43] as this could be another reason for having a variation in the prevalence of mutations.

Our findings have important clinical implications. Firstly, the previously held notion that Arab population has less frequent BRCA mutations compared to other regions of the world [7] may not be entirely true. It is also possible that the BRCA mutations may be linked to the increases in the breast and ovarian cancer cases in Arab countries [29] along with other reasons such as weaker screening programs [44]. Increasing incidences in the breast and ovarian cancer cases in the region could also be associated with having children at older age, increased use of oral contraceptives and reduced breastfeeding as well as increasing level of obesity and physical inactivity [45, 46]. Appropriate risk assessment strategies may help manage and control the risk of breast and ovarian cancer, targeted particularly in the region with higher BRCA mutations [5].

Our study has some limitations. There were paucity of data on BRCA mutations in most part of the Arab world. All of the 14 studies included in this paper came from nine countries and the rest of the 14 countries had no published reports on BRCA prevalence. As for the quality of studies included, most of them had moderate to lower risk of bias, but with higher risk of external validity. This indicates that the studies may not have been well representative of the respective study populations from where these reports originated. There were also substantial heterogeneity among the studies, that presented a major threat to the validity of the pooled estimates. High quality epidemiological studies in Arab populations are, thus warranted.

## Conclusion

We conclude that one in five HBOC patients in the Arab world have BRCA mutations. The BRCA2 is the most prevalent type mutation among breast cancer patients in the Arab region, with an estimated prevalence was 17% with a higher pooled prevalence of BRCA mutations in the Levant Region 28%. When we compared with studies from different parts of the world the Arab world appear to have different profiles with varying BRCA frequencies. High quality epidemiologic studies are warranted to understand the impact of the BRCA gene mutations and their role in breast and ovarian cancer incidence, risk and prognosis in the Arab world that is experiencing a steady increase in the cancer incidence.

## Abbreviations

ASR: Age- standardized mortality rate
BART: Comprehensive BRACAnalysis and BRACAnalysis rearrangement test
BC: Breast cancer
BRCA1: Breast cancer 1 gene
BRCA2: Breast cancer 2 gene
CIS: Carcinoma in situ
CS: Capture sequencing
CSS: Conventional individual exon-by-exon sanger sequencing
DHPLC: Denaturing high performance liquid chromatography
EOC: Epithelia ovarian cancer
HAC: Heteroduplex assay confirmation
HBC: Hereditary breast cancer
HBOC: Hereditary breast or/and ovarian cancer
HOC: Hereditary ovarian cancer
MLPA: Multiple ligation dependent probe amplification
NGS: Next generation sequencing
OV: Ovarian cancer
PCR: Polymerase chain reaction
SS: Sanger sequencing
SSCP: Single strand conformation polymorphism assay
TCS: Targeted capture sequencing
TS: Target screening for exon 10 in BRCA
YLL: Year of life lost
